# Rhabdomyolysis due to the additive effect of statin therapy and hypothyroidism: a case report

**DOI:** 10.1186/1752-1947-1-130

**Published:** 2007-11-10

**Authors:** Ekrem Yeter, Telat Keles, Tahir Durmaz, Engin Bozkurt

**Affiliations:** 1 Department of Cardiology, Ataturk Education and Research Hospital, Ankara, Turkey

## Abstract

We describe a patient with previously undiagnosed hypothyroidism who developed rhabdomyolysis while taking a statin. He had no other precipitating factors. The statin was stopped, intravenous fluids were started immediately and L-thyroxin was given after confirming the diagnosis of hypothyroidism. His symptoms improved over a few days. Because rhabdomyolysis is a rare but potentially life threatening disorder when complicated by acute tubular necrosis and renal failure, physicians must pay special attention when starting statins in patients with hyperlipidemia.

## Introduction

Statins (3-hydroxy-3-methylglutaryl coenzyme A reductase inhibitors) have been widely used as the first choice for treatment of hyperlipidemia. Side effects of statins are relatively infrequent [1]. The most common side effects are skeletal muscle complaints, including clinically important myositis and rhabdomyolysis, mild serum creatine kinase (CK) elevations, myalgia with or without elevated CK, muscle weakness, muscle cramps, and persistent myalgia [2]. The risk of rhabdomyolysis and other adverse effects with statin use can be exacerbated by several factors, including compromised hepatic and renal function, hypothyroidism, diabetes, and concomitant medications [2]. Herein, we report a case of rhabdomyolysis due to atorvastatin in a patient without any precipitating factors other than hypothyroidism.

## Case presentation

A 56-year-old man was admitted to hospital with complaints of a two week history of severe myalgia and proximal muscle weakness of the extremities, with difficulty in exercise and climbing stairs. He denied vigorous physical exercise and alcohol use. His medications consisted of ramipril for hypertension and atorvastatin for hyperlipidemia which he had taken for the previous two weeks. He had no familial or prior personal history of thyroid disease or muscle disorders. He had no previous history of muscular toxicity with statin or fibrate use.

On physical examination, he was afebrile, had periorbital puffiness, lip swelling and mild diffuse goitre, normal heart rate (89 beats/min). All the limbs were swollen and he had pitting edema. Other systems were normal.

On admission, laboratory measurements revealed: hemoglobin 11.3 g/dl, total leucocyte count 7.7 × 109/l, serum K 3,9 mEq/l, Na 137 mEq/l, urea 34 mg/dl, creatinine 1,4 mg/dl blood glucose 85 mg/dl. Serum muscle enzymes were markedly elevated: CK 3471 IU/l (normal up to 170), CK-MB 90 IU/l (normal up to 15), LDH 730 IU/l (150–500), Aspartate transaminase (AST) 91 IU/l, alanine transaminase (ALT) 50 IU/l. Urine analysis showed moderate blood on dipstick, but on microscopic examination there were no erythrocytes. Therefore, we assumed that this was due to myoglobulinuria. Thyroid function tests confirmed the diagnosis of hypothyroidism: thyroid stimulating hormone (TSH) >75 uIU/ml (0.4–4), free T3 (FT3) 0.85 pg/ml (1.57–4.71), free T4 (FT4) 0.3 pg/dl (0,85–1,78). The diagnosis was rhabdomyolysis secondary to the additive effect of hypothyroidism and atorvastatin. Atorvastatin was stopped, intravenous fluids were started immediately and L-thyroxin (100 μg/day) was given after confirming the diagnosis of hypothyroidism. His symptoms progressively improved in a few days. On discharge, two week after admission, serum CK, AST and ALT measurements had decreased to 668 IU/l, 23 IU/l, and 27 IU/l respectively. TSH level was in the normal range 6 weeks after starting treatment.

## Discussion

The report describes a patient with rhabdomyolysis due to the additive effect of undiagnosed hypothyroidism and atorvastatin. Statins have been found to be effective in primary prevention as well as secondary prevention of coronary disease [3]. Although statins are well tolerated by most of the patients, they may cause myopathy, rhabdomyolysis and elevated liver enzymes [4]. Medications that inhibit cytochrome P-450 (CYP) 3A4, such as macrolide antibiotics, antifungals, and cyclosporine, increase serum concentrations of statins and the risk of rhabdomyolysis [2]. There have been several case reports of rhabdomyolysis induced by hypothyroidism [5] but most of the reported cases were precipitated by exercise [6]. In a previous report, 11.7% of patients with primary hypothyroidism accidentally received statins without having the diagnosis of hypothyroidism. Severity of hypothyroidism might be partially associated with elevation of CK. Statins might be a risk factor for severe myopathy and rhabdomyolysis in patients with hypothyroidism [1].

## Conclusion

We wish to alert the physicians to the importance of early recognition and treatment of hypothyroidism before starting statins, which is essential in reducing risk of mortality and complications from rhabdomyolysis. Screening thyroid function before starting statins is important to avoid rare but serious complications.

## Competing interests

The author(s) declare that they have no competing interests.

## Authors' contributions

EY drafted the manuscript. TD participated in the sequence alignment. TK participated in the design and coordination and helped to draft the manuscript. EB is department chair. All authors read and approved the final manuscript.

## Consent

The manuscript was written after obtaining written informed consent from the patient for publication.
